# Circulating miR-181a as a novel potential plasma biomarker for multimorbidity burden in the older population

**DOI:** 10.1186/s12877-022-03451-3

**Published:** 2022-09-29

**Authors:** Francesca Iannone, Paolina Crocco, Serena Dato, Giuseppe Passarino, Giuseppina Rose

**Affiliations:** Department of Biology, Ecology and Earth Sciences, University of Calabria, 87036 Rende, CS Italy

**Keywords:** Aging, miRNA, Multimorbidity, Inflammation, miR-181a, Inflamma-miRs

## Abstract

**Background:**

Chronic low-level inflammation is thought to play a role in many age-related diseases and to contribute to multimorbidity and to the disability related to this condition. In this framework, inflamma-miRs, an important subset of miRNA able to regulate inflammation molecules, appear to be key players. This study aimed to evaluate plasma levels of the inflamma-miR-181a in relation to age, parameters of health status (clinical, physical, and cognitive) and indices of multimorbidity in a cohort of 244 subjects aged 65- 97.

**Methods:**

MiR-181a was isolated from plasma according to standardized procedures and its expression levels measured by qPCR. Correlation tests and multivariate regression analyses were applied on gender-stratified groups.

**Results:**

MiR-181a levels resulted increased in old men, and significantly correlated with worsened blood parameters of inflammation (such as low levels of albumin and bilirubin and high lymphocyte content), particularly in females. Furthermore, we found miR-181a positively correlated with the overall multimorbidity burden, measured by CIRS Comorbidity Score, in both genders.

**Conclusions:**

These data support a role of miR-181a in age-related chronic inflammation and in the development of multimorbidity in older adults and indicate that the routes by which this miRNA influence health status are likely to be gender specific. Based on our results, we suggest that miR-181a is a promising biomarker of health status of the older population.

## Highlights

Levels of the inflamma-miR-181a correlate with multimorbidity burden in older people.

MiR-181a levels correlate with blood inflammation markers in a gender-specific manner.

MiR-181a is positively correlated with age in males but not in females.

The paths by which miR-181a can influence health status likely differ between genders.

## Background

The continuous rise of older individuals in western societies is leading to a significant increase of the prevalence of age-related diseases, such as cardiovascular diseases, diabetes, dementia, and cancer. Alongside this, there has been a significant increase in the prevalence of multimorbidity, i.e., the coexistence of two or more health conditions in an individual, and comorbidity, i.e., the presence of one or more additional conditions concurrent with a primary condition, resulting in a higher proportion of subjects at higher risk of disability, functional loss, and death [1–4]. Currently, inflammaging, the upregulation of the inflammatory response occurring in normal aging process and that determines a chronic, low-grade pro-inflammatory state, is considered one of the central pathogenetic mechanisms at the basis of most diseases and comorbidities accompanying aging [5].

MicroRNAs (miRNAs) are small, non-coding endogenous RNA molecules that regulate gene expression either by mRNA cleavage/destabilization or inhibition of translation [6]. The high capacity of miRNAs to target large networks of mRNAs makes them master epigenetic regulators, able to fine-tune almost all cellular processes [7]. Multiple miRNAs have been demonstrated to regulate canonical aging signalling pathways, and increasingly recognized as important modulators of the processes associated with age-related decline [8–10]. As summarised in several reviews, the dysregulated expression of many of them has been linked to multiple chronic diseases of aging [11–15]. A crucial role in the induction of aging phenotypes seems to be played by a relatively small number of miRNAs involved in the regulation of immune response and inflammatory processes, termed inflamma-miRs [16, 17]. Diverse patterns of dysregulated expression profiles of inflamma-miRs (miR-21, miR-34, miR-126, miR-146a, and miR-155, to name a few) have been identified in pathological conditions [18–20], thus making them attractive biomarkers of the quality of aging.

Mir-181a, a member of the miR-181 family, is increasingly regarded as an inflamma-miR [21] because of its ability to modulate the expression of important anti-inflammatory (TGFβ and IL-10) and pro-inflammatory (IL-6, TNFα, IL-1) cytokines [22, 23], and key components of NF-κB signalling [24]. It also exerts immune modulatory functions mainly by regulating T cell differentiation and proliferation [25–28]. Recent research has also classified miR-181a as a mitochondrial miRNA (“mitomiR”) because its activity in controlling the expression of genes at the crossroads of mitochondrial function, response to inflammation and cellular senescence [21, 29].

The levels of this mediator of inflammation have been correlated with increasing number of chronic diseases, including coronary artery disease [30], neurodegenerative diseases [31] and cancer [31, 32]. Moreover, studies in mice support its role in the loss of muscle mass and function with age [33]. These findings highlight the potential of miR-181a as biomarker of aging and gauge of individual decline. To date, however, few studies have assessed the changes of miR-181a expression levels with age and the relationship with development of negative health outcomes associated to normal aging.

Also, gender differences remain largely unexplored, although this may be a clinically relevant factor considering that the differential expression of miRNAs between the sexes has been found an important underlying mechanism for gender-biased disease outcome [34].

Thus, the aim of this study was to investigate age-and sex-related changes in miR-181a levels in a cohort of subjects in the age-range 65–97 years and to explore the relationship with parameters (clinical, physical, and cognitive) related to the health status and with indices of multimorbidity.

## Methods

### Study participants

Samples used in this study have been collected from elderly nursing homes located in the Calabria region (southern Italy) as part of a larger study examining the quality of aging in Calabria. The study population included 244 subjects with age (range 65–97 years, mean age 82.45 ± 7.11 years) of which 160 were females (range 65–97 years, 83.18 ± 7.12) and 84 males (range 65–97 years, mean age 81.07 ± 6.91 years). A descriptive analysis of this segment of the population, comprising also indicators of their health and functional status, was provided in De Rango et al., 2011 [35]. According to this survey, for its characteristics the studied cohort is representative of the elderly Calabrian population of the same age range. All study participants underwent a multidimensional geriatric assessment designed to evaluate cognitive status, functional abilities, physical health, and social aspects. Data were collected through a structured questionnaire administered by a trained operator. Subjects with physical or mental severe impairments were excluded from the sampling. A peripheral blood sample was collected for each participant for clinical and laboratory examination, and an informed consent was signed. The study was performed following the Strengthening the Reporting of OBservational studies in Epidemiology (STROBE) statement [36].

### Geriatric assessment

#### Physical performance

Muscle strength was assessed as hand grip strength (HGS) using a handheld dynamometer (SMEDLEY’s dynamometer TTM) while the subject was sitting with the arm close to his/her body. The test was repeated three times with the stronger hand and the maximum of these values was considered.

Gait speed was measured at the usual pace over 4-m (m). Timing began when subjects started foot movement and stopped when one foot contacted the ground after completely crossing the 4 m mark. Gait speed was measured using distance in meters and time in seconds (m/s). The best time of two attempts was recorded.

#### Functional activity

Disability was measured using the Activities of Daily Living (ADL) score [37]. The score is given counting the number of activities (bathing, dressing, toileting, transfer from bed to chair, and feeding) in which the participant is dependent or independent at the time of the visit. ADL scores were dichotomized as one if the subject was not independent in all five items and zero otherwise.

#### Cognitive performance

The mini mental state examination (MMSE) tool was used to assess cognitive function, evaluating orientation, episodic memory, attention, language, and construction functions [38]. The MMSE scores (from 0 to 30) were normalized for age and educational status [39]. A MMSE score < 24 was used to diagnose cognitive impairment.

#### Multimorbidity

Multimorbidity was evaluated using the modified Cumulative Illness Rating Scale (CIRS), a scoring system that measures the burden of chronic medical illnesses by considering 14 items, corresponding to different physiological systems (cardiac, vascular, respiratory, otorhinolaryngology, upper gastrointestinal, lower gastrointestinal, hepatic and pancreatic, renal, genitourinary, musculoskeletal and dermatologic, neurology, endocrinology, metabolic, breast, and psychiatric) [40, 41]. The score for each organ system was based on disease severity ranging from 0 (no problem) to 4 (severe impairment in function), resulting in a total score ranging from 0 to 56. Higher scores indicate higher morbidity burden. A comorbidity index was computed by counting the number of items ranking three or four in disease severity.

#### Biochemical measurements

Samples from the venous blood were withdrawn after an overnight fast of 12 h in the morning.

Biochemical measurements were performed at the Italian National Research Centre on Ageing (Cosenza) using standard protocols. The same blood sample was used for miRNA analysis.

#### RNA extraction and miRNA quantification

Plasma for miRNAs analysis was separated by centrifugation at 1800 g for 10 min at room temperature, collected in RNase-free tubes and further centrifuged at 1200 × g for 20 min at 10 °C to completely remove contaminant cells. All samples were kept at − 80 °C until analysis.

MiR-181a was isolated from 200 μL of plasma using miRNeasy® Serum/Plasma kit (Qiagen, Hilden, Germany) according to the manufacturer’s instructions. *Arabidopsis thaliana miR-159a* (assay ID 000,338) was used as a spike-in control throughout the workflow. RNA yield was quantified on the Qubit 2.0 Fluorometer (Life Technologies, Milan, Italy) and it was around 30–50 ng/mL each sample. Of this, 5 μL were converted in cDNA using TaqMan® microRNA Reverse Transcription Kit (Life Technologies) and stem–loop specific RT primers for miR-181a (hsa-miR-181a-5p assay, ID 000,480). Small nuclear (snRNA) U6 was used as endogenous control (assay ID 001,973). Afterward, quantitative real-time PCR was performed on a QuantStudio3™ Real-Time PCR System (Applied Biosystems, Milan, Italy) with automatic baseline setting. All reactions were run in triplicate. The relative expression levels of the miRNA in comparison with the normalizer were then calculated using the comparative threshold (Ct) method 2^−ΔCt^ [42].

### Statistical analysis

Categorical variables are reported as percentages, while continuous variables are reported as means and standard deviations (SD). The Kolmogorov–Smirnov test was used to evaluate the normality of data distribution. Differences in anthropometric and clinical parameters between groups were determined by independent-samples t-test for normal distribution data, the Mann–Whitney U test for skewed data or the Chi-Square test for categorical variables. Pearson’s and Spearman’s correlation tests were used to determine the relationship between parametric and non-parametric variables, respectively. Effect sizes (Cohen's d) were computed using group means with considering the dispersion of the group mean values. Partial correlation was used to test the strength of associations while controlling for age. Multivariate regression analyses were also used to control for potentially confounding factors. Statistical comparisons among groups were performed by using one-way ANOVA followed by post hoc Tukey’s test. All statistical data were analyzed by the SPSS software version 27.0 (SPSS, Inc., Chicago, IL, USA). *P* < 0.05 was considered statistically significant.

## Results

Table 1 summarizes the anthropometric, clinical, and biochemical characteristics of the study subjects, both in the whole sample and in subjects divided according to sex. As Table 1 shows, several characteristics were differently distributed between females and males, but no significant differences in miR-181a mean values were found between the two genders.

**Table 1:** Demographic and selected clinical characteristics

**Variables**	**Entire Cohort** **(***N* = 244**)**	**Female** (*N* = 160)	**Male** **(***N* = 84**)**	***p*-value**	**Effect Size** **(Female vs Male)**	**95% CI**
Age (yrs)	82.45 (7.11)	83.18 (7.12)	81.07 (6.91)	0.026	-0.299	-0.565- -0.034
BMI (Kg/m2)	25.77 (6.06)	25.53 (6.61)	26.32 (4.51)	ns	-	-
Glucose (mg/dL)	99.16 (29.93)	99.29 (31.95)	98.91 (25.91)	ns	-	-
HbA1C (%)	6.08 (1.56)	6.15 (1.63)	5.95 (1.41)	ns	-	-
Total protein (g/dL)	6.58 (0.59)	6.57 (0.59)	6.59 (0.60)	ns		
Albumin (g/dL)	54.61 (8.03)	53.44 (7.67)	56.62 (8.29)	0.010	0.403	0.137–0.670
Total cholesterol (mg/dL)	159.16 (40.44)	163.86 (40.42)	150.50 (39.32)	0.024	-0.334	-0.599- -0.068
LDL cholesterol (mg/dL)	88.75 (32.79)	90.76 (32.53)	85.04 (33.17)	ns	-	-
HDL cholesterol (mg/dL)	49.69 (14.17)	51.39 (14.62)	46.56 (12.81)	0.021	-0.344	-0.610- -0.079
Triglycerides (mg/dL)	116.74	122.65 (71.97)	105.84 (44.21)	ns	-	-
Creatinine (mg/dL)	1.12 (0.43)	1.05 (0.43)	1.24 (0.40)	< 0.0001	0.452	0.185–0.720
Total bilirubin (mg/dL)	0.70 (0.36)	0.67 (0.34)	0.75 (0.38)	ns	-	-
Uric acid (mg/dL)	4.64 (1.39)	4.58 (1.40)	4.78 (1.38)	ns	-	-
Ferritin (ng/mL)	142.50 (130.75)	126.07 (126.43)	170.34 (134.08)	0.004	0.343	0.077–0.609
C-Reactive Protein (mg/L)	14.63 (20.90)	15.30 (22.60)	13.17 (17.07)	ns	-	-
RBC (× 10^12^/L)	4.26 (0.65)	4.20 (0.60)	4.37 (0.73)	0.017	0.263	0.003–0.528
WBC (× 10^9^/L)	6.74 (2.09)	6.91 (2.28)	6.41 (1.64)	ns	-	-
Lymphocytes %	24.64 (10.58)	26.07 (10.67)	22.06 (9.99)	0.012	-0.384	-0.650- -0.118
Neutrophils %	56.57 (16.05)	56.88 (14.48)	55.99 (18.63)	ns	-	-
Monocytes %	10.31 (5.18)	10.22 (4.88)	10.48 (5.70)	ns	-	-
Basophils %	0.83 (0.53)	0.83 (0.53)	0.81 (0.52)	ns	-	-
Eosinophils %	2.55 (2.13)	2.50 (2.13)	2.65 (2.15)	ns	-	-
Platelets (× 10^9^/L)	244.48 (107.28)	262.19 (117.33)	212.73 (77.43)	< 0.0001	-0.469	-0.737—-0.202
HGS (Kg)	17.54 (10.49)	12.53 (5.98)	24.91 (11.36)	< 0.0001	1.504	1.208—1.80
GS Gait Speed (m/s*)*	0.64 (0.28)	0.55 (0.22)	0.78(0.32)	0.001	0.889	0.613—1.165
MMSE	18.01 (5.64)	17.52 (5.87)	18.91 (5.12)	ns	-	-
ADL dependence (> 1)	65.5%	71.8%	53.2%	0.008	0.351	-
CIRS-TS	17.71 (12.57)	17.86 (12.65)	17.38 (12.47)	ns	-	-
CIRS- CI	2.54 (2.90)	2.62 (3.01)	2.37 (2.81)	ns	-	-
Mir-181a (log 2^− ΔCt^)	-0.28 (1.18)	-0.26 (1.22)	-0.30 (1.09)	ns	-	-

Continuous variables are expressed as mean and standard deviations (SD), while categorical variables are expressed as percentage (%). *P* value from t-test or Mann–Whitney depending on continuous data distribution and from chi-squared test of association for categorical variables

*Abbreviations*: *HGS* Hand Grip Strength, *GS* Gait *S*peed, *LDL* Low-density lipoprotein, *HDL* High-density lipoprotein, *RBC* Red Blood Cells, *WBC* White Blood Cells, *MMSE* Mini Mental State Examination, *ADL* Activities of Daily Living, *CIRS-TS* Cumulative Illness Rating Scale (CIRS)-Total Score, *CIRS-CI*, Cumulative Illness Rating Scale (CIRS)-Comorbidity Index, *ns* not significant

### Correlation analysis between miR-181a levels and age

**Fig. 1:**
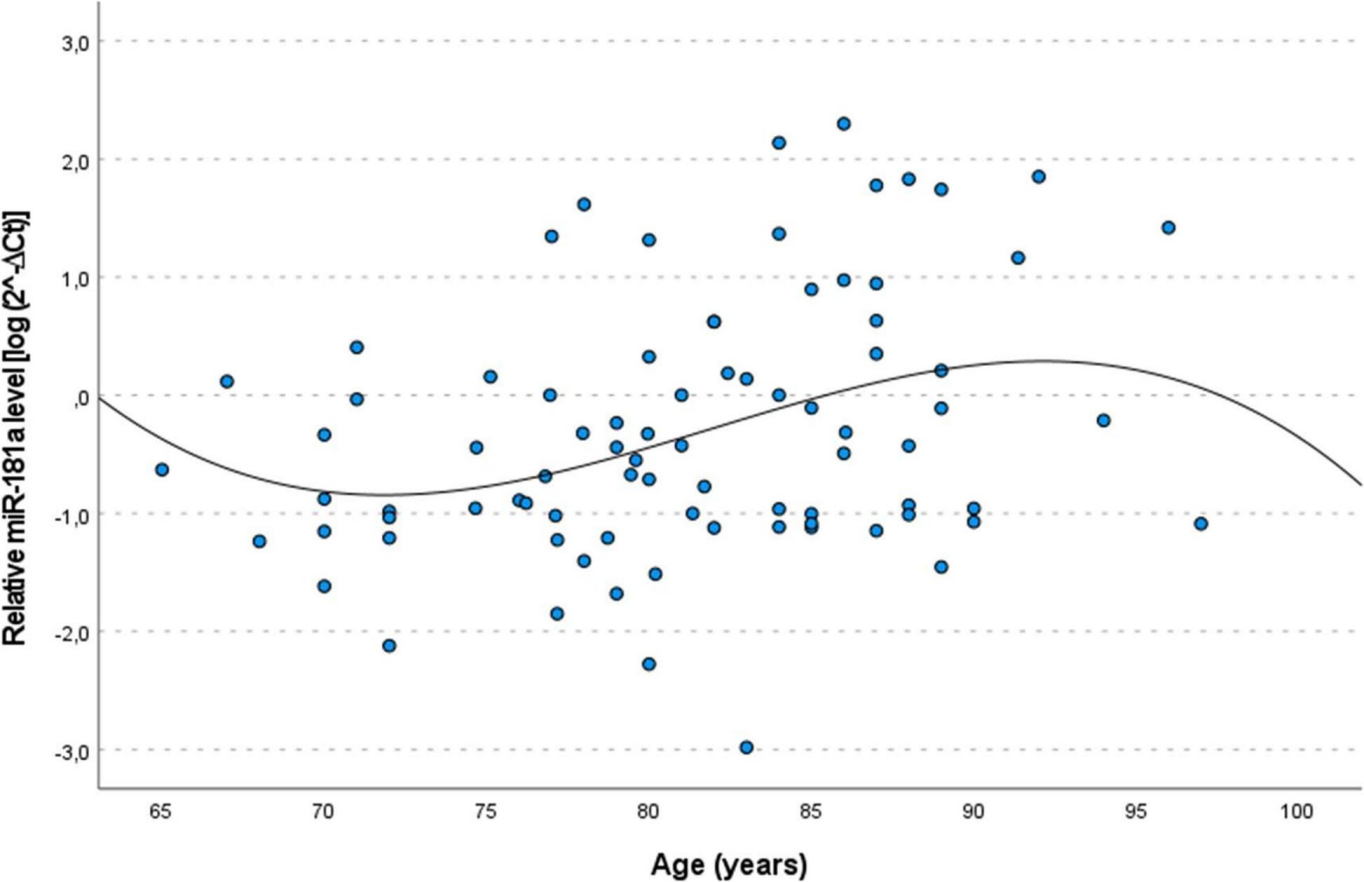
Scatter plot and fitted cubic curve of plasma miR‐181a expression levels as a function of age in male subjects. Data are reported as log 2^− ΔCt^ normalized to U6 expression

First, we investigated changes of miR-181a expression occurring upon age. In the entire cohort, no significant correlation with age was observed (*r* = 0.057, *p* = 0.347). To explore potential sex-specific age-related changes the analysis was conducted separately for women and men, revealing a difference between the two sexes: in males there was a statistically significant increase of miR-181a levels with increasing age (*r* = 0.295, *p* = 0.006) but not in females (*r* = -0.057, *p* = 0.437) This result was confirmed by linear regression analysis [β = 0.312 (95% CI = 0.102–0.522), *p* = 0.004 in males and β = -0.008 (95% CI = -0.164–0.169, *p* = 0.922 in females]. To better delineate the age-related changes, polynomial regression analysis showed that cubic curve fits the data better than a linear curve (linear regression *r*^2^ = 0.098 *vs* cubic regression *r*^2^ = 0.116), indicating a major increase after the age of 80 years (Fig. 1).

Given the sex-specific effect of age on miR-181a expression, we conducted the subsequent analyses with parameters reported in Table 1 separately in the two sexes, after controlling for age.

### Correlation analysis between miR-181a levels and indices of physical, cognitive performance and multimorbidity

We first evaluated the relationship between the miR-181a levels and physical and cognitive abilities measured by geriatric assessments, including Hand Grip (HG), Gait Speed (GS), Activity of Daily Living (ADL), and Mini Mental State Examination (MMSE). No correlation was found between miR-181a levels and these geriatric parameters.

**Fig. 2:**
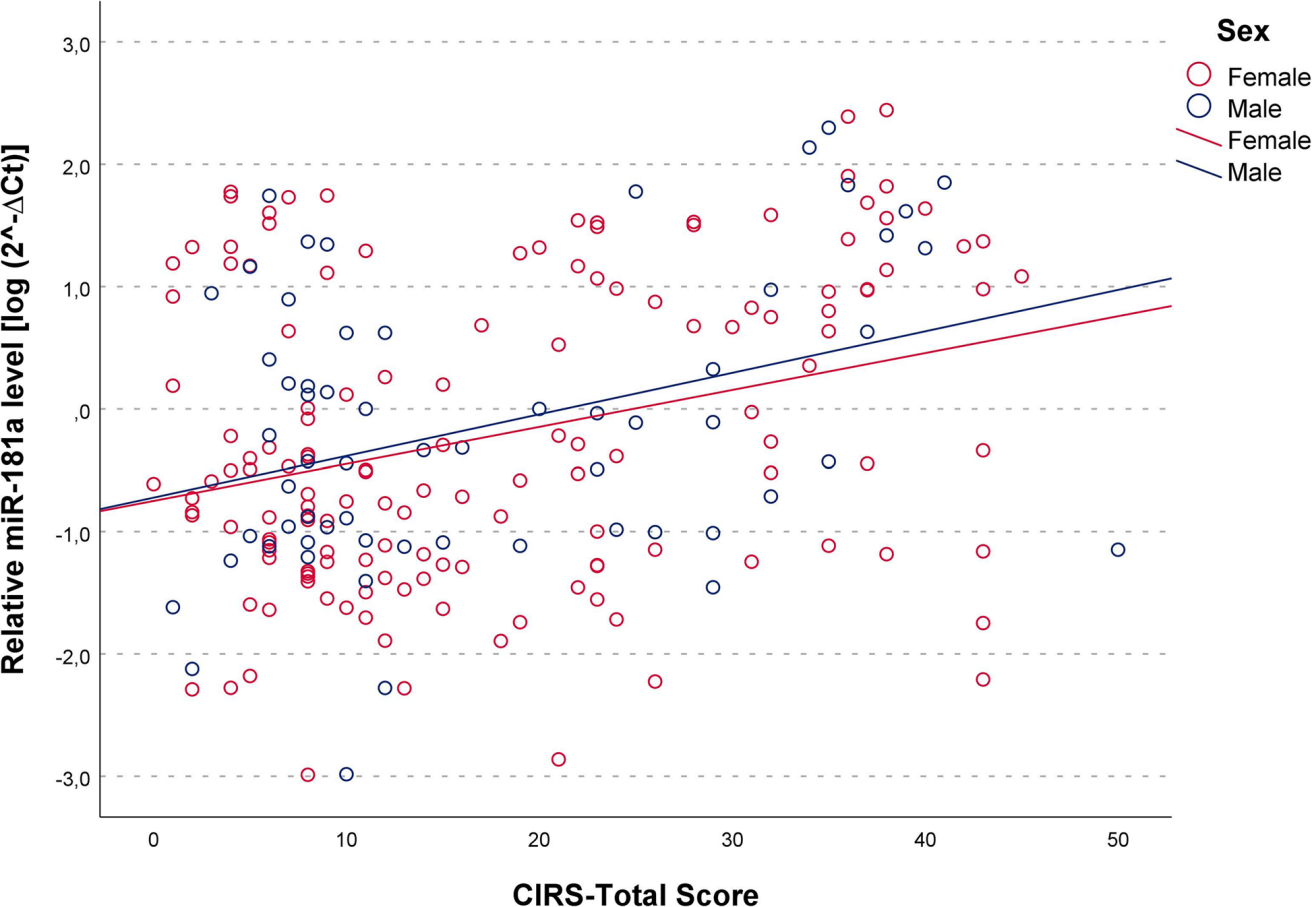
Correlation between plasma miR‐181a levels and multimorbidity. Scatter plots illustrate the relationship between plasma miR‐181a levels and the Cumulative Illness Rating Scale (CIRS) total score in male and female subjects. Data are reported as log 2^− Δ^.^Ct^ normalized to U6 expression. Regression lines are displayed for males (blue, r_partial_ = 0.306, *p* = 0.026) and females (red, r_partial_ = 0.306, *p* = 0.026)

We then sought to examine the relationship between levels of miR-181a and multimorbidity burden by using the Cumulative Illness Rating Scale for Geriatrics (CIRS-total score) index. We found this index positively correlated with age of subjects (*r* = 0.268, *p* = 0.001 and *r* = 0.277, *p* = 0.03 for female and males respectively). Partial correlation analysis adjusted for age showed that circulating levels of miR-181a were significantly higher in subjects with a higher multimorbidity burden (that is, higher CIRS total score) in both sexes (r_partial_ = 0.307, *p* < 0.001 and r_partial_ = 0.330, *p* = 0.016, in women and men respectively). Linear regression analysis confirmed the positive correlation between miR-181a expression and CIRS after the adjustment of age [β = 0.330 (95% CI = 0.176–0.503), *p* < 0.001 and β = 0.299 (95% CI = 0.046–0.602), *p* = 0.023, respectively for females and males]. The scatter plots and linear regressions relative to these tests are presented in Fig. 2. Similarly, evaluation of the CIRS-Comorbidity Index (CIRS-CI), computed by counting the number of items for which moderate to severe pathology was reported, yielded positive correlations [r_partial_ = 0.292, *p* = 0.001 and β = 0.289 (95% CI = 0.127–0.483), *p* = 0.001] in females and [r_partial_ = 0.306, *p* = 0.026 and β = 0.302 (95% CI = 0.043–0.639) *p* = 0.026] in males, again reflecting higher miR-181a levels among persons with greater multimorbidity.

### Correlation analysis between miR-181a levels and clinical markers

**Fig. 3:**
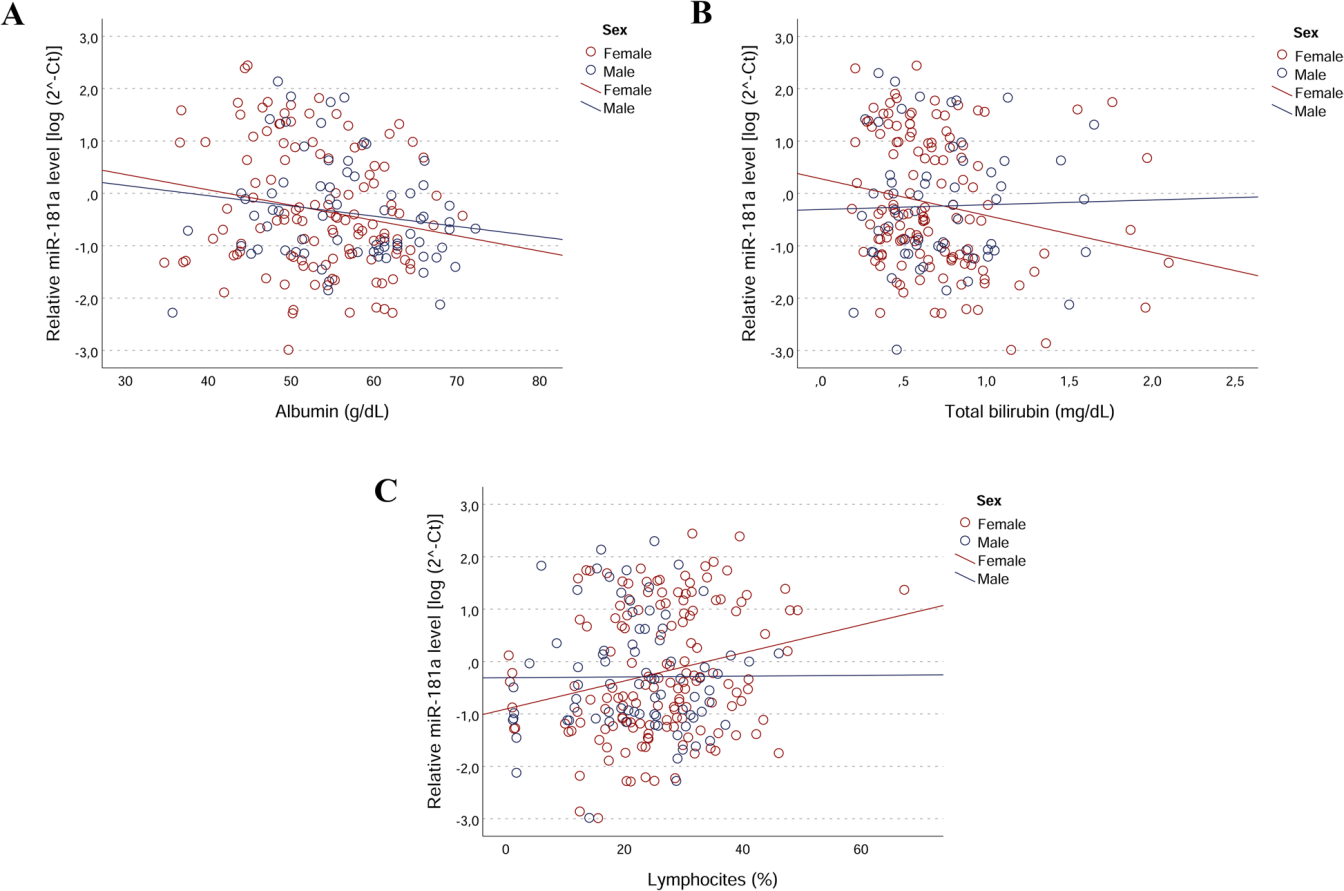
Correlations between plasma miR‐181a levels and biochemical variables in male and female subjects. Scatter plots illustrate the relationship between plasma miR‐181a levels and levels of (**A**) Albumin ( *p* = 0.041 in females); (**B**) Total Bilirubin (*p* = 0.024 in females); (**C**) Lymphocytes (*p* = 0.01 in females). Data are reported as log 2^− ΔCt^ normalized to U6 expression

The association of miR-181a with the health status of analysed samples prompted us to investigate potential associations with clinical measurements. Again, sex-specific associations between miR-181a levels and blood biomarkers of generalized inflammation were found. More precisely, in women miR-181a expression was negatively correlated with Albumin [r_partial_ = -0.187; *p* = 0.041 and β = -0.188 (95% CI = -0.354—-0.008, *p* = 0.041] and total bilirubin [r_partial_ = -0.199; *p* = 0.024 and β = -0.199 (95% CI = -0.383—-0.028, *p* = 0.024] levels, and positively correlated with lymphocytes percentage [r_partial_ = 0.213; *p* = 0.004 and β = 0.237 (95% CI = 0.097—0.401, *p* = 0.004]. Scatter plots and linear regressions are shown in Fig. 3 (A, B and C). In males, miR-181a levels tended to be positively correlated with neutrophils percentage, but this correlation did not reach statistical significance at nominal level.

## Discussion

In the present study, circulating levels of miR-181a, widely regarded as an inflamma-miR [16, 21], have been analysed in a cohort of elderly subjects aged from 65 to 97 years. We found that in both genders, but more significantly in females, circulating miR-181a levels positively correlated with the burden of multimorbidity when assessed either by the CIRS total score or by the CIRS-Comorbidity Index, which are reliable measures of multimorbidity and poor health status in geriatric patients [41, 43]. Multimorbidity is recognized as the most common chronic condition, determining a progressive increase of susceptibility to the occurrence of functional impairment and disability [44]. Factors affecting its development are complex and manifold [45], but chronic inflammation has consistently been found as one of its main risk factors [46]**.**

The positive correlation of miR-181a with the burden of multimorbidity which we report, considering the role of miR-181a as a modulator of inflammatory responses, suggests that higher expression of miR-181a may be associated with a higher inflammatory state.

Interestingly, increased levels of miR-181a have been reported in inflammatory- and oxidative stress-related conditions, such as after treatment with lipopolysaccharide and hydrogen peroxide [47, 48]. Also, the overexpression of this miRNA has been seen in diverse age-related illnesses, including neurodegenerative and cancer diseases, although contrasting results have been found in the direction of the effect of miR-181a on these diseases [30–32, 49, 50]. These divergent results can be reconciled by considering that miR-181a can play a dual role as an anti- or a pro-inflammatory miRNA, likely depending on the cellular and physiological context.

Of note, we found miR-181a levels negatively correlated with the levels of albumin and bilirubin and positively correlated with lymphocytes in women, while levels in men only showed a trend toward a positive correlation with neutrophils. Although these findings must be interpreted with caution as some of the correlations only approach statistical significance, they may provide some hints about the relationship among miR-181a, inflammation and health status. First, they are compatible with our previous suggestion that the upregulation of miRNA-181a expression correlates with inflammatory status, given that low levels of albumin and bilirubin and high levels lymphocytes and neutrophils are characteristic features of inflammation [51–54]. Second, sex-specific correlations may reflect specific differences in immune-inflammatory responses between men and women with age. For example, men older than 65 years display higher innate and lower adaptive immune function than women [55, 56]. Being lymphocytes and neutrophils among the main cell types involved in the adaptative and innate response, respectively, the association of miR-181a levels with lymphocytes in females and neutrophils in males could mirror these differences.

The finding that miR-181a is positively correlated with age in males but not in females further supports this view. Studies investigating age associated changes of miR-181a expression in vitro and in vivo models yielded contrasting results, with some reports showing a downregulation [57, 58] and others an upregulation [29, 59]. As to humans, two studies found miR-181a downregulated in older individuals compared to younger ones [22, 60]. However, the subjects analysed in the above cited studies were younger than those included in our study (< 70 years). In addition, sex-stratified analyses were not performed in those studies, thus making it challenging to compare results and draw a conclusion. On the other hand, mir-181a has been found up-regulated in centenarians [61, 62]. These evidences, together with ours, suggest that changes in miR-181a levels with age follow nonlinear trajectories, characterized by a decrease in the expression levels from young to old age, and by an increase from old to very old age. This reasoning is supported by literature reports of nonlinear age-related changes and sex-dependent differences in levels of various miRNAs, including miR-181a [34, 63, 64], as well as non-linear alterations in plasma proteome with age [65].

## Conclusion

Data herein support the hypothesis that miR-181a acts as an inflamma-miRNA playing important roles in the aging process. We were able to identify miR-181a as multimorbidity-associated miRNA. Its potential use as biomarker of health status has to consider that it might have different effects at different ages, as well as reported for other factors [66, 67]. Importantly, our results highlighted sex-specific correlations of miR-181a with risk factors for negative outcomes, suggesting that the routes by which this miRNA can influence health status are different between genders. From a more general point of view, this supports the growing belief that for better understanding the underpinnings of the gender differences in aging, as well as in age-related diseases, gender-stratified analyses should be performed.

Our conclusions should be viewed in the light of some limitations. First, the correlations we found are of moderate effect, partially due to the relatively small size of the analysed sample; thus, a larger sample would have probably allowed us to better document the validity of our statistical associations. Therefore, larger future studies and across a wider age range are recommended. Second, the results here presented are limited to revealing associative, rather than causal, relations between miR-181a expression and multimorbidity at old age. Therefore, investigations on its targets and regulators would provide more insights about the signalling pathways underlying the observed associations and evaluate its potential as biomarker of multimorbidity risk.

Notwithstanding these limitations, data here presented may lay the groundwork for a more complete research study in the future.

## Data Availability

The data that support the findings of this study are available from the corresponding author upon reasonable request.

## Abbreviations

miRNA: MicroRNA
qPCR: Quantitative Polymerase Chain Reaction
CIRS: Cumulative Illness Rating Scale
inflamma-miRs: Inflammatory microRNAs
TGFβ: Transforming Growth Factor beta
IL-10: Interleukin 10
IL-6: Interleukin 6
TNFα: Tumor necrosis factor alfa
IL-1: Interleukin 1
HGS: Hand Grip Strength
ADL: Activities of Daily Living
MMSE: Mini Mental State Examination
cDNA: Complementary DNA
SD: Standard Deviation
BMI: Body Mass Index
HbA1C: Haemoglobin A1C
GS: Gait Speed
LDL: Low-density lipoprotein
HDL: High-density lipoprotein
RBC: Red Blood Cells
WBC: White Blood Cells
CIRS-TS: Cumulative Illness Rating Scale (CIRS)-Total Score
CIRS-CI: Cumulative Illness Rating Scale (CIRS)-Comorbidity Index
